# AI-driven genotype-phenotype modeling: a framework integrating multi-modal single-cell genomics and reverse vaccinology for *de novo* design of multi-epitope cancer vaccines

**DOI:** 10.3389/fgene.2026.1909167

**Published:** 2026-09-11

**Authors:** Mahabub Mallik, Sahid Afrid Mollick

**Affiliations:** 1 Department of Data Science, The West Bengal National University of Judicial Science, Kolkata, West Bengal, India; 2 Anthropological Survey of India, Head Office, Kolkata, West Bengal, India

**Keywords:** artificial intelligence, generative AI, genotype-phenotype modeling, multi-epitope vaccine design, multi-modal single-cell genomics, personalized cancer immunotherapy, reverse vaccinology

## Abstract

Cancer vaccines have emerged as a promising strategy for personalized cancer immunotherapy; however, their development has traditionally relied on bulk sequencing approaches that average molecular information across millions of cells, thereby obscuring the extensive intratumoral heterogeneity that drives disease progression, therapeutic resistance, and immune escape. Recent advances in multi-modal single-cell genomics have transformed the ability to characterize tumors at unprecedented resolution, enabling the identification of distinct cellular populations, clonal evolutionary trajectories, and complex tumor-immune interactions. In parallel, artificial intelligence (AI) has rapidly expanded the capabilities of reverse vaccinology by facilitating large-scale analysis of genomic and immunological datasets for neoantigen discovery and vaccine design. This review aims to present a unique conceptual framework for future personalized cancer immunotherapies, rather than simply integrating the already established approaches. The framework is built on two levels: (1) filtering of false-positive targets using multi-modal single cell data and removing antigen loss clones; and (2) feeding the resulting rigorously filtered data into advanced structural and generative AI models to inform *de novo* design of multi-epitope vaccines. Particular emphasis is placed on the application of deep learning, graph neural networks, transformer architectures, and generative AI models for data preprocessing, clonal evolution analysis, immune microenvironment characterization, neoantigen prioritization, and peptide–major histocompatibility complex (MHC) interaction prediction. Furthermore, we discuss the development of integrated computational pipelines capable of translating high-resolution multi-modal single-cell data into personalized multi-epitope cancer vaccines. Finally, we highlight the major translational challenges, including model interpretability, tumor plasticity, manufacturing constraints, and clinical implementation. By integrating multi-modal single-cell genomics with advanced AI methodologies, reverse vaccinology is poised to accelerate the development of highly targeted, adaptive, and durable cancer vaccines, offering a promising roadmap for the future of personalized cancer immunotherapy.

## 1 Introduction

Cancer remains one of the leading causes of morbidity and mortality worldwide, imposing an enormous clinical, social, and economic burden on healthcare systems. Despite remarkable advances in diagnosis and treatment, the complexity and heterogeneity of malignant diseases continue to challenge effective long-term management. Conventional therapeutic modalities, including surgery, radiotherapy, and chemotherapy, have significantly improved patient survival. However, these approaches often suffer from limited specificity, systemic toxicity, and the eventual emergence of treatment resistance (Hanahan, 2022). As a result, there has been a growing shift toward precision oncology, an approach that seeks to tailor therapeutic interventions according to the unique molecular characteristics of an individual’s tumor (Ashley, 2016). This narrative mini-review is based on a synthesis of peer-reviewed literature on multi-modal single-cell genomics, reverse vaccinology, artificial intelligence, multi-epitope vaccines design and personalized cancer immunotherapy. Priority was given to recent primary research articles, landmark studies, and selected high-quality reviews that provide mechanistic insights into neoantigen discovery, immunogenicity prediction, AI-assisted computational frameworks, and clinical translation. The selected literature was critically evaluated to highlight current advances, existing challenges, and future directions in AI-guided personalized cancer vaccine development.

In recent years, cancer immunotherapy has emerged as one of the most transformative developments in oncology. Unlike traditional treatments that directly target tumor cells, immunotherapy harnesses the patient’s immune system to recognize and eliminate malignant cells. Immune checkpoint inhibitors, adoptive cell therapies, and therapeutic cancer vaccines have demonstrated the remarkable potential of immune-based interventions in several cancer types (Ribas and Wolchok, 2018). Nevertheless, durable responses remain limited to a subset of patients, largely due to the extensive genetic diversity of tumors and their ability to evade immune surveillance through dynamic evolutionary processes (McGranahan and Swanton, 2017). Among the various immunotherapeutic strategies, therapeutic cancer vaccines have attracted substantial interest because of their ability to induce highly specific and long-lasting anti-tumor immune responses.

The application of reverse vaccinology to cancer is different in its very nature compared to the traditional application to infectious diseases, and mechanistically more complex (Saxena et al., 2021). Although vaccinology for infectious diseases is based on the identification of stable, genome-derived microbial antigens, cancer vaccinology must also take into consideration the dynamic nature of somatic clonality, individual HLA restriction, decreased tumor antigen processing, dynamic immune editing and active suppression of the tumor microenvironment.

Neoantigens arise from somatic mutations that generate novel peptide sequences absent from normal tissues. Because these mutated peptides are recognized as non-self by the immune system, they represent highly attractive targets for personalized immunotherapy (Schumacher and Schreiber, 2015). However, identifying clinically relevant neoantigens remains a formidable challenge due to the enormous number of mutations present within tumors and the complex biological processes governing antigen processing, presentation, and immune recognition.

The concept of reverse vaccinology has emerged as a powerful framework to address these challenges. Originally developed for infectious disease vaccine discovery, reverse vaccinology inverts the traditional vaccine development paradigm by beginning with genomic information rather than laboratory-based antigen screening (Rappuoli, 2000). Through computational analysis of genomic and proteomic datasets, candidate antigens can be identified, prioritized, and optimized entirely *in silico* before experimental validation. In the context of cancer, reverse vaccinology enables systematic screening of tumor-derived mutations to identify neoantigens with the highest potential to elicit effective immune responses (Feng et al., 2025). This computational approach significantly accelerates vaccine development while reducing the costs and limitations associated with conventional antigen discovery methods.

Recent advances in artificial intelligence (AI) and machine learning have further expanded the capabilities of reverse vaccinology. The explosive growth of multi-omics data has created unprecedented opportunities to apply advanced computational models capable of identifying patterns beyond the scope of traditional statistical approaches. Deep learning architectures, transformer-based models, graph neural networks (GNNs), and large language models (LLMs) are increasingly being utilized to predict peptide immunogenicity, major histocompatibility complex (MHC) binding affinity, antigen processing efficiency, and T-cell receptor interactions (Winnifrith et al., 2024; Chowell et al., 2021). By integrating vast quantities of genomic, transcriptomic, structural, and immunological information, these AI-driven frameworks can prioritize clinically relevant neoantigens with greater accuracy and speed than conventional prediction algorithms (Navin et al., 2011). The implementation of these workflows will require lots of compute power to process multi-modal single-cell data, and rapid-response decentralized GMP manufacturing nodes to mitigate unacceptable time-to-treatment for late-stage patients. In addition, new regulatory routes will need to be developed, because the current regulatory process was designed to approve vaccine batches for offline, static drugs, not for online, changing AI algorithms that adapt vaccine formulations according to the real-time evolution of the tumors.

Despite these advances, a critical limitation remains: most neoantigen discovery pipelines rely heavily on bulk sequencing technologies. Bulk sequencing provides an averaged molecular profile derived from millions of cells, effectively masking the extensive cellular heterogeneity that characterizes most solid tumors (Dagogo-Jack and Shaw, 2017). Tumors are not homogeneous masses but rather dynamic ecosystems composed of genetically distinct sub-clonal populations that continuously evolve under selective pressures imposed by the immune system and therapeutic interventions (Thol et al., 2022). Consequently, neoantigens identified through bulk sequencing may fail to capture clinically important minority clones that contribute to disease progression, metastasis, and treatment resistance.

Single-cell genomics has emerged as a transformative technology capable of overcoming these limitations. By enabling molecular profiling at the resolution of individual cells, single-cell sequencing technologies provide unprecedented insights into intratumoral heterogeneity, clonal evolution, and the complex interactions between tumor cells and their surrounding microenvironment (Gawad et al., 2016). Single-cell RNA sequencing (scRNA-seq), single-cell DNA sequencing (scDNA-seq), single-cell ATAC sequencing (scATAC-seq), and single-cell immune repertoire profiling allow researchers to reconstruct tumor evolutionary histories, identify rare but clinically significant sub-clonal populations, and characterize immune cell states with remarkable precision (Kuipers et al., 2017). Such high-resolution information offers a more comprehensive and biologically meaningful foundation for neoantigen discovery and personalized vaccine development.

The convergence of multi-modal single-cell genomics and artificial intelligence therefore represents a promising next-generation framework for precision cancer immunotherapy. Single-cell technologies provide the detailed biological context necessary to understand tumor evolution, while AI-driven reverse vaccinology offers the computational power required to translate this information into actionable therapeutic designs. scDNA-seq overcomes the antigen-loss problem by identifying clonally conserved mutations that are more likely to remain stable therapeutic targets. scRNA-seq establishes an expression threshold to actively exclude mutations that are not transcriptionally expressed, thereby reducing false-positive neoantigen candidates. scATAC-seq helps design effective cancer vaccines by identifying the specific open DNA regions and master regulators in tumor cells that drive immune evasion. scTCR-seq identifies pre-existing T-cell receptor repertoires and their activation or exhaustion states, enabling prioritization of neoantigens that are more likely to elicit effective immune responses. Circulating tumor DNA (ctDNA) can complement these single-cell approaches by enabling longitudinal monitoring of clonal evolution, treatment response, and the emergence of new neoantigens, thereby supporting adaptive refinement of personalized cancer vaccine strategies during disease progression. Together, these complementary data layers create the possibility of developing highly personalized, multi-epitope cancer vaccines capable of targeting both dominant and emerging tumor clones while adapting to the dynamic nature of cancer progression (Baslan and Hicks, 2017; Winnifrith et al., 2024; Sahin et al., 2017).

This review explores the emerging integration of multi-modal single-cell genomics and AI-guided reverse vaccinology as a unified strategy for personalized cancer vaccine development. Particular emphasis is placed on the role of single-cell technologies in resolving intratumoral heterogeneity, the application of advanced AI models for genotype-phenotype prediction and neoantigen prioritization, and the development of integrated computational pipelines capable of accelerating vaccine design. Finally, the review discusses the major translational challenges, including model interpretability, manufacturing constraints, and clinical implementation, that must be addressed before these technologies can be routinely deployed in precision oncology.

## 2 Precision oncology and the need for multi-modal single-cell AI-Guided reverse vaccinology

### 2.1 The crux of precision oncology

The ultimate goal of precision oncology is to shift the paradigm of cancer treatment away from the “one-size-fits-all” approach, like conventional cytotoxic chemotherapies, to more molecularly targeted and patient-specific therapies. The basic principle of this paradigm is that cancer is not a single disease, but an extremely personalized evolutionary process, which is shaped by specific somatic changes (Wu and Xie, 2025). Through high-throughput diagnostic sequencing, doctors hope to detect a person’s specific genetic changes that can be used to treat a cancer (Sharma and Goel, 2025). This genomic blueprint is then used to choose targeted therapies that are matched to the patient and/or to create personalized immunotherapies, like therapeutic vaccines. Recent studies further demonstrate that integrating circulating genomic biomarkers with artificial intelligence–assisted analytical frameworks can improve prediction of treatment resistance and immunotherapy response, thereby facilitating more precise patient stratification and personalized therapeutic decision-making in oncology (Wang et al., 2025).

### 2.2 The bottleneck of bulk genomics

In spite of the historical advances that have been brought by the next-generation sequencing, bulk tissue genomics is a huge hurdle for the realization of precision medicine. Bulk sequencing is based on the concept of ‘blender’ – it is a method that merges millions of various tumor cells, stromal cells and immune cells together to get a single average genomic profile (Pirouzbakht et al., 2025). This macro-level perspective does not reveal intratumoral heterogeneity, the presence of many different sub-clonal populations of malignant cells which contribute to tumor evolution (Le et al., 2025). As a result, interventions based on bulk genomic data are often incomplete and targets are selected, clones evade the treatment, and patients relapse.

### 2.3 The dawn of AI-guided reverse vaccinology

To overcome these limitations and maximize the potential of personalized immunotherapies, the field is seeing the dawn of artificial intelligence (AI)-guided reverse vaccinology (RV). Reverse vaccinology is a method that uses the genomic information of a pathogen or tumour to identify and design antigens *in silico*, avoiding the time-consuming conventional biochemical screening approach (Mahmud and Banerjee, 2026). AI-driven RV can combine cutting-edge computational intelligence techniques, like deep learning, graph neural networks (GNNs), and transformer models, to analyze vast amounts of data and forecast hyper-accurately which types of mutations in tumor cells will generate highly immunogenic neoantigens (Das et al., 2026). Unlike standard algorithms that typically model linear, non-biological features, AI algorithms can model complex, non-linear biological features, like peptide processing pathways, proteasomal cleavage, and the highly polymorphic binding affinities between patient-specific peptides and major histocompatibility complex (MHC) molecules, which can dramatically shave the timeline from tumor biopsy to vaccine formulation.

### 2.4 Conceptual framework of multi-modal single-cell AI-RV

Multi-modal single-cell genomics can resolve the intratumoral heterogeneity and trace the evolution of individual clones in an accurate manner, thus offering a true, uncontaminated picture of the genetic makeup of a malignancy. This high-resolution information can, when coupled with predictive and generative AI frameworks, be used to precisely prioritize and design multi-epitope vaccines targeting both dominant and emerging sub-clonal populations (Vohra et al., 2025). This paper provides an overview (but not comprehensive either) of the various components in this new blueprint, ranging from single-cell data pre-processing, through the tracking of a single cell’s sub-clones, to structural modelling of peptide-MHC interactions using AI, with an eye towards the challenges that remain in the transition to clinical usage of this automated, intelligent pipeline.

## 3 Multi-modal single-cell genomics: Overcoming heterogeneity and mapping clonal evolution

### 3.1 Deconvoluting intratumoral heterogeneity

Malignant tumors do not consist of identical cells, but are highly complex mosaic ecosystems with unique cell sub-populations. Traditional genomics cannot detect these signals, and averages them out, effectively obscuring minority clones. Single-cell RNA sequencing (scRNA-seq), single-cell ATAC sequencing (scATAC-seq), and single-cell DNA sequencing (scDNA-seq) overcome this limitation by providing different molecular profiles for thousands of single cells at once (Madan et al., 2025). Such deconvolution yields a better understanding of cellular heterogeneity within the tumor and can separate malignant cells from infiltrating non-malignant cells, map different cell transcriptional states, and isolate rare, highly aggressive cell phenotypes (Ozcelik et al., 2024). The granular decomposition means that individual targets for treatment are chosen according to the detailed mathematical analysis of the composition of the tumor and not just by a mean value of the tissue.

### 3.2 Tracking clonal and sub-clonal evolution

Multi-modal single-cell somatic mutation data can be used to build up the high-resolution phylogenetic trees that represent the evolution of a tumor from its founding clone to the many sub-clonal branches (Oseni et al., 2025). The tracking of the clonal architecture guarantees that selected neoantigens would target both trunk mutations (shared by all cells), and branch mutations (shared by the most important sub-clones) and thus would effectively contain the tumor and curb mechanisms of clonal escape.

### 3.3 Profiling the tumor immune microenvironment

The effectiveness of a cancer vaccine based on artificial intelligence (AI) depends greatly on the TME (tumor microenvironment) in the tumor’s vicinity. Single-cell genomics allows for comprehensive characterization of the stromal, myeloid-derived suppressor and tumor infiltrating lymphocytes that influence local immune responses (Biswas and Chakrabarti, 2020). Single-cell TCR (T-cell receptor) sequencing, combined with transcriptomics, can be used to identify if a patient’s T-cell are actively expanded or exhausted, or are suppressed in function. Profiling this landscape will determine if a personalized vaccine will need to be formulated with specific adjuvants, or combined with immune checkpoint inhibitors, in order to overcome local immunosuppression, and to promote a strong cytotoxic response against the tumor (Patel et al., 2025).

### 3.4 AI-driven preprocessing and data harmonization

Pre-processing models powered by AI processes this messy data, transforming it into a tidy environment for vaccine development. At this stage, deep learning architectures are very powerful, such as Variational Autoencoders (VAEs), including scVI(Mahmud and Banerjee, 2026). These neural networks are trained to learn a low dimensional continuous latent representation of the data obtained from the single cell.

## 4 AI-driven genotype-phenotype modeling in reverse vaccinology

### 4.1 Deep learning for variant annotation and prioritization

Targetable tumor neoantigens start with a population of somatic mutations that are identified from single-cell data, followed by only a small percentage of these mutations being immunologically relevant. Automation of the cumbersome process of variant annotation and prioritization is enabled by deep learning models such as phylogenetic machine learning frameworks (Souleiman and Souleiman, 2026). These neural networks consume genomic sequences and come up with predictions of the functional impact of nonsynonymous single-nucleotide variants (SNVs) and insertions/deletions (indels) (Ozturk et al., 2026). Using the parameters of evolutionary conservation, changes in the structure of the resulting protein, and sub-clonal frequency data, the AI searches through thousands of mutations, and prioritizes those most likely to be stably expressed and most different from the host immune system’s own profile.

### 4.2 Modeling peptide-MHC interaction

The major hurdle of reverse vaccinology is to identify if a modified peptide would be readily processed and presented by the very polymorphic Human Leukocyte Antigen (HLA) system of the patient. Recently, this deep learning architecture of genotype-phenotype modeling has been transformed by deep learning models such as Transformers and Graph Neural Networks (GNNs). GNNs are direct representations of the mutated peptide and the HLA binding groove in 3D, showing the spatial atomic interactions and chemical properties of the peptide. At the same time, transformer models incorporate attention mechanisms to account for long range amino acid dependencies in the sequence (Al-Kabani et al., 2025). These models, coupled with one another, have unprecedented predictive power for proteasomal cleavage, TAP transport efficiency, and peptide-MHC binding affinity, without the need for tedious biochemical assays.

### 4.3 Predicting immunogenicity and T-cell recognition

While the identification of tumor-specific mutations and prediction of peptide–major histocompatibility complex (MHC) binding represents critical steps in cancer vaccine development, they do not necessarily guarantee an effective immune response. A substantial proportion of predicted neoantigens are capable of binding MHC molecules yet fail to elicit meaningful T-cell activation. Consequently, one of the greatest challenges in modern reverse vaccinology is distinguishing merely presented peptides from truly immunogenic neoantigens capable of triggering robust anti-tumor immunity (Schumacher and Schreiber, 2015).

Immunogenicity is a multifaceted biological phenomenon governed by a complex interplay between antigen presentation, T-cell receptor (TCR) recognition, immune-cell activation, and the broader tumor microenvironment. For a neoantigen to become an effective vaccine target, it must not only be processed and displayed on the surface of tumor cells but also be recognized as foreign by T-cell with sufficient affinity to initiate an immune response (Yadav et al., 2014). This process is further complicated by immune tolerance mechanisms, antigen escape strategies employed by tumors, and the extensive diversity of human leukocyte antigen (HLA) alleles across populations (McGranahan and Swanton, 2017).

Traditional computational pipelines have largely focused on predicting peptide–MHC binding affinity as a surrogate marker of immunogenicity. Although such approaches have significantly accelerated neoantigen discovery, accumulating evidence suggests that MHC binding alone is an insufficient predictor of T-cell responses (Wells et al., 2020). Many strongly binding peptides fail to induce immune activation, whereas certain peptides with moderate binding affinities can generate potent anti-tumor responses. These observations have highlighted the need for more comprehensive predictive frameworks capable of modeling the entire antigen recognition process.

Recent advances in artificial intelligence and machine learning have substantially improved the ability to predict neoantigen immunogenicity. Deep learning models can integrate diverse biological features, including peptide sequence composition, antigen-processing efficiency, MHC presentation probability, TCR recognition patterns, gene-expression levels, and structural characteristics of peptide–MHC complexes (Chowell et al., 2021; Bulashevska et al., 2024; Mei et al., 2019). By learning from large repositories of experimentally validated epitopes, these algorithms can identify subtle patterns associated with successful immune activation that may be overlooked by conventional statistical approaches.

A particularly promising area involves the application of transformer-based architectures and protein language models (PLMs), which adapt concepts from natural language processing to biological sequence analysis. By treating amino acid sequences as biological languages, PLMs learn contextual and evolutionary relationships among residues from millions of protein sequences. These representations capture structural and functional information that can be leveraged to improve prediction of antigen presentation, peptide immunogenicity, T-cell receptor recognition, and other determinants of immune response (Rives et al., 2021; Winnifrith et al., 2024; O'Brien et al., 2024). Similarly, structural AI approaches have benefited from advances in protein-folding prediction, allowing researchers to model peptide–MHC–TCR interactions at unprecedented resolution. Such methods provide valuable insights into the molecular determinants of immune recognition and may help identify neoantigens with enhanced therapeutic potential (Jumper et al., 2021).

The emergence of single-cell technologies has further strengthened immunogenicity prediction by enabling direct characterization of T-cell responses within the tumor microenvironment. Single-cell RNA sequencing and T-cell receptor sequencing can reveal clonal expansion patterns, activation states, exhaustion signatures, and antigen-specific immune responses at the individual-cell level (Zhang et al., 2018). Integrating these datasets with AI-based predictive frameworks creates an opportunity to move beyond theoretical neoantigen identification toward the discovery of functionally validated targets that are actively recognized by patient-derived immune cells.

Despite considerable progress, significant challenges remain. Most predictive models are trained on relatively limited datasets, and experimentally validated cancer neoantigens remain scarce compared with the enormous diversity of potential tumor mutations. Furthermore, differences in HLA composition, tumor type, and immune status can influence model performance and limit generalizability across patient populations (Bulik-Sullivan et al., 2018). Improving the interpretability, robustness, and clinical validation of AI-driven immunogenicity models will therefore be essential for their successful integration into personalized vaccine development pipelines.

Looking ahead, the convergence of artificial intelligence, structural biology, and single-cell immunogenomics is expected to transform the prediction of T-cell recognition from a largely probabilistic exercise into a more precise and mechanistic science. By accurately identifying neoantigens capable of eliciting durable and clinically relevant immune responses, these technologies have the potential to substantially improve the efficacy of next-generation personalized cancer vaccines and advance the broader goals of precision oncology (Papalexi and Satija, 2017; Ott et al., 2017).

### 4.4 Generative AI and *de novo* immunogen design

The next Frontier of AI-driven reverse vaccinology is represented by generative artificial intelligence (AI) and large language models (LLMs) trained on the “language” of proteins to facilitate *de novo* immunogen design. Unlike conventional approaches that assemble naturally occurring peptide fragments, generative models can design artificial multi-epitope vaccine constructs optimized for antigen organization, linker composition, structural stability, and processing efficiency (Wang et al., 2026). While predictive AI models, including graph neural networks (GNNs) and transformer architectures, have demonstrated promising performance in peptide–major histocompatibility complex (MHC) binding and immunogenicity prediction, their broader clinical application remains constrained by the limited availability of experimentally validated training datasets, imbalance in HLA allele representation, insufficient standardized benchmark datasets, and a lack of comprehensive external validation (Villanueva-Flores et al., 2025). In contrast, generative LLMs remain largely experimental, and computationally designed vaccine candidates require rigorous biological and clinical validation before therapeutic application. Nevertheless, these emerging technologies provide a promising strategy for the rational design of compact, structurally stable, and multi-epitope vaccine constructs capable of eliciting broad, durable, and polyfunctional T-cell responses against the dynamic clonal landscape of tumors.

### 4.5 Comparative assessment of AI methodologies

The diverse AI methodologies currently employed in reverse vaccinology differ substantially in their biological objectives, computational maturity, and current limitations. Rather than representing competing approaches, these methods should be viewed as complementary components of an integrated neoantigen discovery pipeline. Conventional deep learning models are primarily used for variant prioritization and immunogenicity prediction, graph neural networks (GNNs) facilitate structural modeling of peptide–major histocompatibility complex (MHC) interactions, transformer architectures and protein language models (PLMs) improve sequence representation and immunogenicity prediction, whereas generative AI supports *de novo* multi-epitope vaccine design. Despite encouraging advances, no single AI methodology currently addresses all stages of personalized cancer vaccine development. A comparative summary of the principal AI methodologies currently applied in reverse vaccinology is presented in Table 1.

**TABLE 1:** Comparative assessment of major AI methodologies used in reverse vaccinology for personalized cancer vaccine development.

AI methodology	Primary application	Major strengths	Evidence-supported limitations/failure modes	Current maturity
Deep neural networks (DNNs)	Variant annotation, peptide processing, immunogenicity prediction	Capture complex nonlinear biological relationships and integrate heterogeneous molecular features	Limited interpretability (“black-box”), require large high-quality labelled datasets, susceptible to overfitting when training data are limited	Widely used in computational research; limited prospective clinical validation (Shafiei et al., 2026)
Graph neural networks (GNNs)	Structural modelling of peptide–HLA interactions and molecular representation learning	Naturally model structural and relational information; improve molecular interaction prediction	Performance depends on graph quality and construction; limited interpretability; limited external benchmarking and cross-dataset generalizability	Experimental/emerging (Corso et al., 2024)
Variational autoencoders (VAEs)	Latent-space sequence encoding, bioactive peptide generation, and continuous representation learning for epitope/peptide optimization	Map discrete biological sequences into continuous latent representations; enable smooth interpolation, probabilistic sampling, and property-guided candidate generation	Generated sequences may contain non-functional or biologically implausible candidates; performance depends on training-data quality and latent-space representation; posterior collapse can occur; candidates require rigorous structural, physicochemical, and biological validation	Emerging/experimental, with proof-of-concept validation in peptide and protein design (Dean and Walper, 2020; Greener et al., 2018)
Generative Adversarial networks (GANs)	*De novo* peptide sequence design, synthetic data augmentation for limited immunogenicity datasets, and structural molecular design	Generate realistic sequence candidates and expand limited training datasets through synthetic sample generation; activity-aware GANs can incorporate desired biological properties during generation	Vulnerable to mode collapse and unstable adversarial training; generated sequences may lack desired binding or biological activity without appropriate constraints and filtering; synthetic data may inherit training-set biases; extensive downstream validation is required	Emerging/experimental, with proof-of-concept applications in bioactive peptide generation and *de novo* peptide/protein design (Tucs et al., 2020; Lin et al., 2022)
Transformer models	Peptide sequence modelling, HLA-binding prediction, immunogenicity prediction	Capture long-range sequence dependencies; highly scalable; effective through transfer learning	Depend on large training datasets and computational resources; reduced performance for underrepresented HLA alleles and out-of-distribution data; limited explainability	Rapidly emerging with increasing experimental validation (Machaca et al., 2024; Consens et al., 2025)
Protein language models (PLMs)	Protein representation learning, neoantigen prioritization	Learn contextual protein representations from millions of sequences and improve downstream prediction accuracy	May inherit training-data bias; biological interpretability remains limited; many downstream applications require additional experimental validation	Emerging (Leclercq and Droit, 2025)
Generative AI/Large language models (LLMs)	*De novo* multi-epitope vaccine and protein sequence design	Enable rational sequence generation, linker optimization and rapid exploration of candidate vaccine constructs	Most applications remain experimental; generated candidates require extensive biological validation; absence of standardized benchmarking and clinical validation	Early-stage/largely conceptual (Wang et al., 2026; Koh et al., 2025)

Overall, current evidence suggests that AI methodologies should be regarded as complementary rather than mutually exclusive. Integrating deep learning, structural graph learning, transformer-based sequence modeling, protein language models, and generative AI with multimodal single-cell genomics has the potential to improve neoantigen prioritization and personalized cancer vaccine design. However, broader clinical translation will require standardized benchmarking, improved model interpretability, external validation across diverse patient cohorts, and prospective biological validation of AI-predicted neoantigens. Recent comparative assessments of large multimodal AI models in oncology further emphasize that, despite substantial advances in AI-assisted clinical decision support, widespread clinical implementation requires rigorous external validation, improved model interpretability, and standardized benchmarking before routine adoption (Huang et al., 2025). Consistent with these requirements, recent clinical studies have demonstrated that integrating artificial intelligence with multi-omics analyses can substantially enhance cancer diagnosis, molecular subtyping, prognostic stratification, and clinical decision-making. Importantly, these studies also emphasize that successful clinical implementation depends on rigorous validation across independent cohorts, functional biological verification, and demonstration of clinical utility before routine adoption, highlighting that computational advances alone are insufficient for clinical translation (Teng et al., 2025; Liu et al., 2025a).

## 5 The integrated multi-modal single-cell AI-RV pipeline

### 5.1 Nature of the proposed framework

**FIGURE 1:**
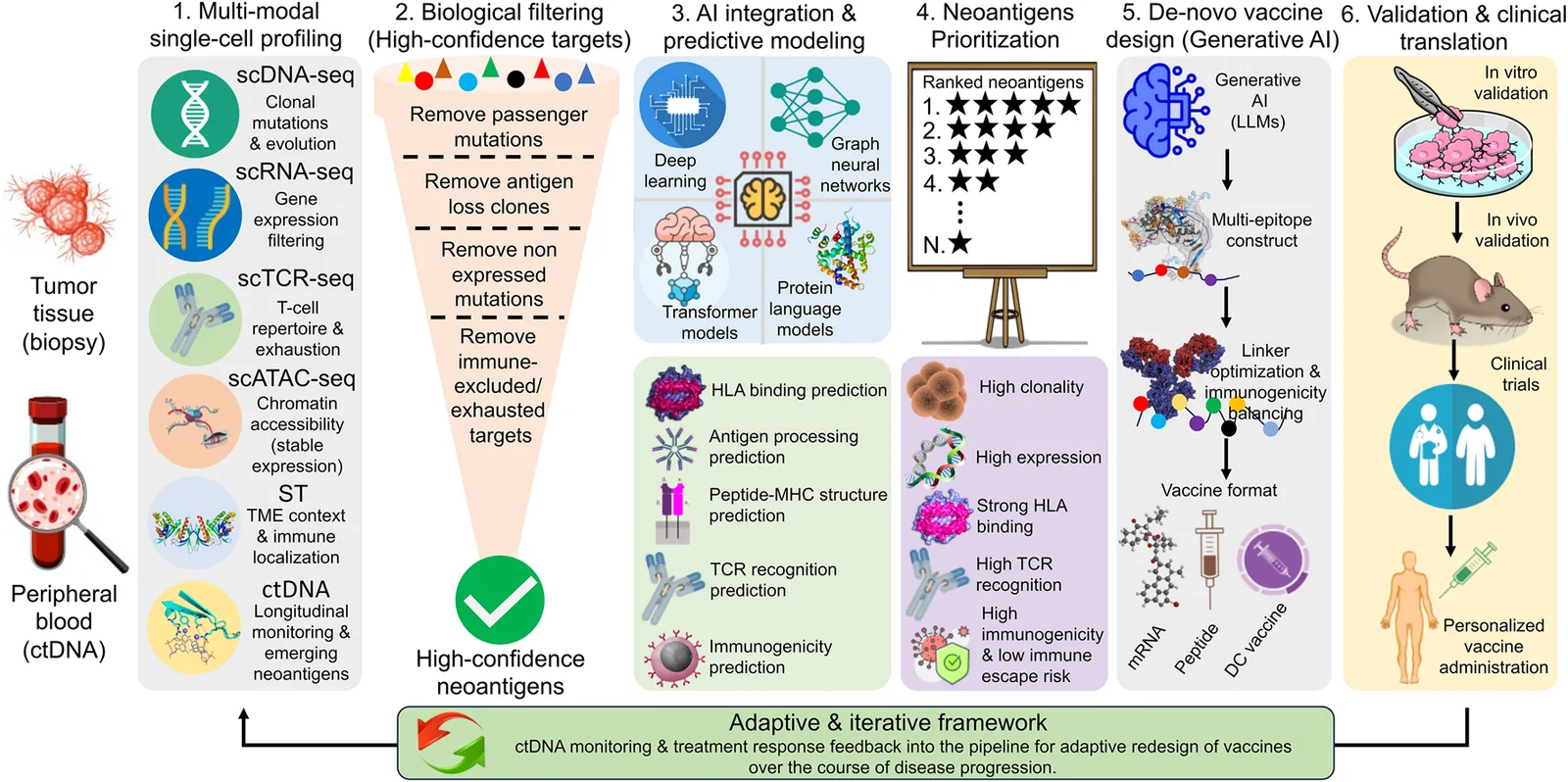
Conceptual framework for multi-modal single-cell AI-guided reverse vaccinology for personalized cancer vaccine development. The framework integrates multi-modal single-cell profiling, biologically informed target filtering, AI-based predictive modelling, neoantigen prioritization, generative AI-assisted *de novo* vaccine design, and experimental/clinical validation within an adaptive framework incorporating longitudinal tumour monitoring.

The workflow presented in this section should be regarded as a conceptual framework that integrates recent advances in multi-modal single-cell genomics, artificial intelligence, and reverse vaccinology into a unified strategy for personalized cancer vaccine development. Although individual components of this workflow—including single-cell sequencing, phylogenetic reconstruction, HLA-binding prediction, immunogenicity prediction, and personalized vaccine manufacturing, have each been independently developed and, in some cases, experimentally or clinically validated, their integration into a single end-to-end AI-driven platform has not yet been comprehensively demonstrated in routine clinical practice. Accordingly, the framework described here should not be interpreted as an established clinical pipeline. Rather, it provides a systems-level blueprint illustrating how complementary technologies could be combined to improve neoantigen prioritization, reduce false-positive candidate selection, and support the rational design of personalized multi-epitope cancer vaccines. We therefore distinguish between currently established methodologies, proposed computational integrations, and future translational opportunities, recognizing that prospective biological validation, standardized benchmarking, regulatory evaluation, and multicenter clinical studies will be essential before such an integrated workflow can be routinely implemented in precision oncology (Figure 1).

### 5.2 High-dimensional data acquisition and deep pre-processing

Within this proposed conceptual framework, the workflow begins with the acquisition of high-resolution genotypic data from single-cell RNA sequencing (scRNA-seq), single-cell ATAC sequencing (scATAC-seq), and single-cell DNA sequencing (scDNA-seq) (Figure 1). This reflects the unprocessed molecular composition of all parts of the tumor ecosystem, including infiltrating immune cells and malignant sub-clones (Dhiman et al., 2009). Variational Autoencoders (VAEs) are used to automatically denoise the data, impute missing values in the transcription (dropouts) and correct for batch effects.

### 5.3 Phylogenetic inference and target prioritization

The somatic mutation data is fed into phylogenetic machine learning algorithms to assemble the evolutionary history of the tumor and trace the evolution of the dominant founder clones as well as the evolution of therapy-resistant sub-clonal lineages (Hou et al., 2025). The variant pool is then passed to the structural AI models, called Graph Neural Networks (GNNs) and Transformers. These models are able to conduct complex genotype/phenotype mapping and determine how well the patient’s particular HLA alleles can bind to and present these mutated peptides (Fang et al., 2026b). The pipeline combines sub-clonal prevalence and MHC binding prediction to yield a very prioritized list of strong targetable neoantigens.

### 5.4 AI-based neoantigen selection and validation

Following phylogenetic reconstruction and preliminary neoantigen prioritization, an additional validation stage is required to identify the most clinically relevant vaccine targets. Although computational analyses may identify hundreds of candidates neoantigens, only a small fraction is capable of eliciting robust and durable anti-tumor immune responses. Therefore, artificial intelligence plays a critical role in refining the candidate pool and selecting high-confidence neoantigens for vaccine development. Modern AI frameworks integrate multiple layers of biological information, including mutant allele frequency, gene expression levels, clonality, peptide-processing efficiency, HLA-binding affinity, and predicted T-cell receptor (TCR) recognition potential. Deep learning models trained on experimentally validated epitope datasets can identify complex patterns associated with successful immune activation that are often missed by conventional scoring methods (Wells et al., 2020). Rather than relying solely on peptide-MHC binding predictions, these systems evaluate the complete antigen presentation and immune recognition pathway.

Recent advances in transformer-based protein language models and structural learning algorithms have further improved immunogenicity prediction. These models can capture long-range sequence dependencies and characterize the molecular interactions among neoantigens, HLA molecules, and T-cell receptors. In parallel, integration of single-cell transcriptomic and TCR sequencing data enables validation of whether specific neoantigens are associated with active, expanded, or exhausted T-cell populations within the tumor microenvironment (Zhang et al., 2018).

The outcome of this multi-layered validation process is a refined panel of highly immunogenic neoantigens that are biologically relevant, clonally conserved, and likely to induce effective anti-tumor responses. These validated targets provide the foundation for constructing personalized multi-epitope cancer vaccines.

### 5.5 Multi-epitope vaccine construction

In the proposed framework, the final computational component involves translating prioritized neoantigens into a candidate multi-epitope vaccine design. The prioritized neoantigen list is fed into the Generative AI, which uses Large Language Models (LLMs) suitable for protein engineering. The AI designs a complex vaccine construct, with multiple epitopes, which is much more efficient than a human having to manually string them together. It rationalizes the spatial organization of the antigens, enables incorporation of desired linker sequences that are non-immunogenic at the junctions and guarantees biochemical stability of the final molecule (Slavny et al., 2024). The final product of this multi-aspect pipeline is a patient-specific vaccine formula that is synthesized and ready for quick manufacturing and later translative deployment.

### 5.6 Clinical translation and manufacturing workflow

Once a personalized multi-epitope vaccine construct has been computationally designed, the focus shifts from *in silico* prediction to real-world clinical implementation. The selected vaccine sequence is synthesized using an appropriate delivery platform, such as messenger RNA (mRNA), synthetic long peptides, dendritic-cell formulations, or viral-vector systems. Among these technologies, mRNA platforms have emerged as particularly attractive due to their rapid manufacturing timelines, scalability, and flexibility for personalized vaccine production (Sahin et al., 2017).

A major challenge in clinical translation is minimizing the time between tumor sampling and vaccine administration. The complete workflow involves tumor biopsy collection, single-cell sequencing, computational analysis, AI-guided neoantigen selection, vaccine design, quality-control testing, and Good Manufacturing Practice (GMP)-compliant production. Because tumors continue to evolve during this period, reducing turnaround time is essential to ensure that the final vaccine remains relevant to the patient’s current tumor landscape (Ott et al., 2017).

Manufacturing personalized vaccines also presents unique regulatory and logistical challenges. Unlike conventional pharmaceuticals, each vaccine is designed for an individual patient and must meet strict quality, safety, and reproducibility standards. Consequently, GMP-compliant manufacturing facilities must incorporate rigorous quality-control procedures, including sequence verification, sterility assessment, purity testing, and potency evaluation before clinical administration (Verbeke et al., 2019).

The successful integration of artificial intelligence, rapid sequencing technologies, and automated manufacturing systems has the potential to significantly accelerate personalized cancer vaccine development. Future advances in decentralized manufacturing facilities, real-time tumor monitoring, and adaptive vaccine redesign may enable dynamically updated immunotherapies capable of responding to ongoing tumor evolution. Nevertheless, the integrated workflow described in this review should be regarded as a proposed conceptual framework rather than a fully validated end-to-end clinical pipeline. While many of its individual components, including single-cell sequencing, AI-assisted neoantigen prioritization, peptide–HLA interaction prediction, and personalized vaccine manufacturing—have been independently developed and, in some cases, experimentally or clinically validated, their integration into a unified AI-driven workflow has not yet been comprehensively validated. Therefore, translating this framework from experimental research to routine clinical practice will require improvements in model interpretability, mitigation of training-data bias and HLA-associated bias, standardized benchmarking, prospective biological validation of computationally predicted neoantigens, streamlined GMP-compliant manufacturing, and rigorous multicenter clinical evaluation.

### 5.7 Comparison with existing neoantigen discovery pipelines

Several computational pipelines have been developed to facilitate personalized neoantigen discovery, including pVACtools (Hundal et al., 2020), NeoPredPipe (Schenck et al., 2019), Vaxrank (Rubinsteyn et al., 2018), OpenVax (Kodysh and Rubinsteyn, 2020), and neoANT-HILL (Coelho et al., 2020). These platforms have substantially advanced computational cancer vaccine development by integrating somatic mutation detection, HLA typing, peptide–major histocompatibility complex (MHC) binding prediction, gene-expression filtering, and neoantigen prioritization into standardized computational workflows. Their application has accelerated personalized cancer vaccine research and supported translational immunotherapy studies by enabling systematic identification of candidate neoantigens from next-generation sequencing data.

Despite these advances, most currently available pipelines rely primarily on bulk whole-exome sequencing (WES) and bulk RNA sequencing, which average molecular signals across heterogeneous tumor cell populations. Consequently, they have limited ability to resolve intratumoral heterogeneity, distinguish trunk from branch mutations, identify antigen-loss clones, characterize tumor-reactive T-cell repertoires, or incorporate spatial information from the tumor microenvironment into neoantigen prioritization. Furthermore, artificial intelligence within existing pipelines is generally limited to peptide–MHC binding prediction or immunogenicity scoring, whereas comprehensive multimodal integration of single-cell datasets remains uncommon.

The conceptual framework proposed in this review extends these established computational pipelines by incorporating complementary single-cell sequencing modalities before AI-driven neoantigen prioritization. Rather than replacing existing tools, the proposed framework introduces biologically informed filtering steps that progressively refine candidate selection. Specifically, scDNA-seq identifies clonally conserved trunk mutations and excludes unstable subclonal variants; scRNA-seq removes mutations lacking transcriptional evidence; scTCR-seq prioritizes neoantigens associated with active tumor-reactive lymphocyte populations while identifying exhausted immune repertoires; scATAC-seq evaluates chromatin accessibility associated with stable antigen expression; and spatial transcriptomics/CITE-seq provide additional information regarding immune-cell localization and regional immune suppression. These biologically filtered candidates are subsequently prioritized using advanced deep learning models, graph neural networks (GNNs), transformer architectures, protein language models, and structural artificial intelligence before optional generative AI-assisted multi-epitope vaccine design.

Importantly, the workflow described here should be regarded as a conceptual systems architecture rather than a clinically validated end-to-end computational pipeline. Although individual components-including single-cell sequencing, AI-assisted neoantigen prediction, and generative vaccine designhave each demonstrated considerable promise independently, their seamless integration into a unified clinical workflow has not yet been experimentally validated. Future benchmarking against established neoantigen discovery platforms together with prospective biological validation and multicenter clinical studies will be essential to determine whether multimodal single-cell integration provides measurable improvements in neoantigen prioritization and personalized cancer vaccine efficacy.

Compares representative neoantigen discovery pipelines with the conceptual single-cell AI-guided reverse vaccinology framework proposed in this review, highlighting differences in biological data integration, artificial intelligence methodologies, and translational scope (Table 2).

**TABLE 2:** Comparison between representative neoantigen discovery pipelines and the conceptual framework proposed in this review.

Feature	pVACtools	NeoPredPipe	Vaxrank	OpenVax	neoANT-HILL	Proposed single-cell AI-RV framework
Primary sequencing input	Bulk WES/WGS + RNA-seq	Bulk WES/WGS	Bulk WES + RNA-seq	Bulk sequencing	Bulk WES + RNA-seq	scDNA-seq + scRNA-seq + scTCR-seq + scATAC-seq + spatial transcriptomics/cite-seq
Somatic mutation detection	✓	✓	✓	✓	✓	✓
HLA typing	✓	✓	✓	✓	✓	✓
Peptide–MHC binding prediction	✓	✓	✓	✓	✓	✓
Gene-expression filtering	Bulk RNA-seq	Bulk RNA-seq	Bulk RNA-seq	Limited	Bulk RNA-seq	Single-cell transcriptomics
Clonal evolution analysis	Limited	Limited	No	No	No	Single-cell phylogenetic reconstruction
Subclonal neoantigen prioritization	Limited	No	No	No	No	Yes
Tumor immune microenvironment integration	No	No	No	No	No	Integrated
scTCR repertoire integration	No	No	No	No	No	Yes
Spatial transcriptomics/CITE-seq	No	No	No	No	No	Optional
AI-based multimodal integration	Limited	Limited	Limited	Limited	Limited	Deep learning + GNNs + Transformers + protein language models
Generative AI-assisted vaccine design	No	No	No	No	No	Conceptual integration
Adaptive vaccine redesign	No	No	No	No	No	Future direction
Current validation status	Translational research	Research	Clinical evaluation	Research	Research	Conceptual framework (not clinically validated)

Summarizes the complementary biological contribution of individual single-cell modalities to neoantigen prioritization and illustrates how multimodal integration can reduce false-positive candidate selection while improving biological confidence (Table 3).

**TABLE 3:** Contribution of individual single-cell modalities to neoantigen prioritization.

Single-cell modality	Biological information obtained	False-positive targets removed	Contribution to vaccine development
scDNA-seq	Somatic mutations, clonality, copy-number alterations	Passenger mutations and unstable subclones	Prioritizes conserved trunk neoantigens
scRNA-seq	Gene-expression profiles	Non-expressed mutations	Selects transcriptionally active neoantigens
scTCR-seq	T-cell clonotypes, clonal expansion, exhaustion	Immunologically irrelevant targets	Prioritizes neoantigens recognized by active T-cell
scATAC-seq	Chromatin accessibility	Epigenetically silenced targets	Improves confidence in stable antigen expression
Spatial transcriptomics	Tumor architecture and immune-cell localization	Immune-excluded regions	Incorporates spatial immune context
CITE-seq	Surface protein expression	RNA-only false-positive candidates	Confirms antigen presentation potential
Single-cell proteomics	Protein abundance and post-translational modifications	Translational artifacts	Supports functional validation of neoantigens

Collectively, the principal distinction between the proposed framework and currently available neoantigen discovery platforms is not the replacement of existing computational algorithms but the integration of biologically informative single-cell datasets throughout the neoantigen selection process. Existing pipelines efficiently prioritize candidate peptides primarily on sequence-derived characteristics, whereas the framework proposed here incorporates tumor evolution, transcriptional activity, immune-cell dynamics, chromatin accessibility, and spatial tumor organization before AI-assisted prioritization and vaccine design. Consequently, this framework should be considered a conceptual roadmap for future development rather than a validated clinical pipeline, providing a foundation for future benchmarking studies and experimental validation aimed at improving precision cancer vaccine development.

## 6 Translational challenges and future perspectives

### 6.1 The “black box” dilemma and explainability

The main computational hurdle to clinical use is the “black box” property of complex deep learning and generative models. Although Graph Neural Networks (GNNs) and Large Language Models (LLMs) can create highly optimized, multi-epitope vaccines, they do not explicitly explain how they came up with the vaccine formulations (Liu et al., 2025b). High stakes medical decisions cannot be left to opaque algorithms, for they are required to be approved and administered by regulatory bodies such as the FDA or EMA, and treating oncologists (Krzyszczyk et al., 2018). The field will need to devise Explainable AI (XAI) frameworks that help to identify the process that the model takes to make its decision, including describing exactly which molecular features were used to select a given neoantigen *versus* another, and that outputs are biologically plausible and clinically substantiated.

However, one of the most important obstacles to clinical use is the reproducibility and external validation of the models, since technical noise and dropout events in single cells significantly affect the identification of neoantigens. To address this, there needs to be standardized experimental protocols and strong, multi-cohort biological validation that a neoantigen that is shown to be promising by AI in one institutional dataset is able to generate the modeled immune response *in vivo* in a variety of patients.

### 6.2 Data quality and standardization

The performance of any artificial intelligence system is fundamentally dependent on the quality of the data used for training and prediction. In the context of single-cell genomics and AI-guided reverse vaccinology, data quality remains one of the most significant barriers to clinical implementation. Single-cell sequencing datasets are often affected by technical noise, batch effects, sequencing errors, dropout events, and variability introduced by differences in sample collection, processing protocols, and sequencing platforms (Lähnemann et al., 2020). Such inconsistencies can influence downstream analyses, including clonal reconstruction, neoantigen discovery, and immunogenicity prediction.

The challenge becomes even greater when integrating datasets generated across different institutions and patient populations. Variations in sequencing depth, library preparation methods, bioinformatic pipelines, and annotation standards can produce conflicting results and reduce the reproducibility of AI models. As a result, a neoantigen identified as highly promising in one dataset may not be prioritized in another, despite representing the same biological phenomenon (Hicks et al., 2017).

Addressing these challenges requires the development of standardized experimental protocols, harmonized bioinformatic workflows, and robust quality-control procedures. Recent advances in AI-based batch correction and data integration methods have improved the ability to combine heterogeneous datasets while preserving biologically meaningful signals. However, establishing universally accepted reference datasets, benchmarking frameworks, and reporting guidelines will be essential for ensuring reproducibility and comparability across studies (Stuart and Satija, 2019).

As personalized cancer vaccines move closer to clinical adoption, data standardization will become increasingly important. Reliable and high-quality datasets are necessary not only for accurate vaccine design but also for building trust among clinicians, regulatory agencies, and patients. Ultimately, the success of AI-guided reverse vaccinology will depend as much on data quality as on the sophistication of the algorithms themselves.

### 6.3 Turnaround time and GMP manufacturing bottlenecks

The proposed pipeline involves obtaining a biopsy, conducting high throughput single cell sequencing, running complex artificial intelligence-based calculations, and physically producing the new vaccine (usually using mRNA or synthetic long peptides) to GMP standards (Siddiqui et al., 2025). This “vein-to-vein” turnaround time is now several weeks to months. In patients with very aggressive late-stage cancers the delay is clinically unacceptable. Sequencing protocols need to be streamlined and decentralized, rapid response GMP manufacturing facilities need to be established to make this pipeline available to the patients who need it most.

### 6.4 Dynamic tumor plasticity and trial design

The multi-modal single-cell data used to sequence the tumor can change dramatically in a few weeks, especially if the patient is on bridging chemotherapy, and it will take several more weeks for the personalized vaccine to be made. This dynamic plasticity could make a vaccine developed at the tumor “Day 0″redundant by “Day 60”. In addition, conventional clinical trial designs and RECIST (Response Evaluation Criteria in Solid Tumors) criteria do not account for the special mechanism of action of immunotherapies, such as pseudo progression, in which tumors grow in size before reducing in size (Wang et al., 2024). To surmount this challenge, clinical trials should be implemented in an adaptive manner, accompanied by AI-RV, which tracks clonal evolution in real-time through circulating tumor DNA (ctDNA).

### 6.5 Regulatory and ethical considerations

The integration of artificial intelligence, multi-modal single-cell genomics, and personalized vaccine design introduces a range of regulatory and ethical challenges that must be addressed before widespread clinical implementation can be achieved. Unlike conventional therapeutics, AI-guided cancer vaccines rely on continuous interaction between genomic data generation, computational decision-making, and individualized manufacturing processes. This complexity creates unique concerns regarding transparency, accountability, patient privacy, and regulatory oversight (Topol, 2018).

One of the primary ethical issues relates to the handling of large-scale genomic datasets. Single-cell sequencing generates highly detailed molecular information that may reveal sensitive genetic characteristics of patients. Ensuring secure data storage, controlled access, and compliance with privacy regulations is therefore essential to protect patient confidentiality and maintain public trust (Shabani and Marelli, 2019). Furthermore, the use of cloud-based computational infrastructures and international data-sharing initiatives raises additional concerns regarding data ownership, governance, and informed consent.

Regulatory agencies face the challenge of evaluating not only the final vaccine product but also the computational algorithms used during vaccine design. Traditional approval frameworks were developed for fixed pharmaceutical products and may not be fully applicable to adaptive AI-driven systems that continuously learn from new data. Consequently, new regulatory approaches may be required to assess algorithmic transparency, reproducibility, robustness, and clinical validity throughout the vaccine-development process (Benjamens et al., 2020).

Another important consideration is algorithmic bias. AI models trained on datasets that inadequately represent diverse populations may generate predictions that perform unevenly across different ethnic, genetic, or geographical groups. Such biases could unintentionally contribute to disparities in access to personalized medicine and compromise clinical outcomes for underrepresented populations (Gianfrancesco et al., 2018). Continuous validation across diverse cohorts will therefore be necessary to ensure equitable healthcare delivery.

Despite these challenges, appropriate regulatory frameworks and ethical governance mechanisms can facilitate the safe and responsible deployment of AI-guided cancer vaccines. Collaboration among clinicians, computational scientists, ethicists, regulatory authorities, and patient advocacy groups will be essential for establishing standards that balance innovation with patient safety. As precision oncology continues to evolve, ethical and regulatory considerations must remain central to the development of next-generation personalized immunotherapies.

## 7 Future directions

The integration of multi-modal single-cell genomics and artificial intelligence (AI)-driven reverse vaccinology represents only the beginning of a broader transformation in personalized cancer immunotherapy. Although current approaches have demonstrated considerable promise in neoantigen discovery and vaccine design, several emerging technologies are expected to further enhance the precision, adaptability, and clinical applicability of personalized cancer vaccines in the coming years.

One of the most promising developments is the emergence of foundation models for biology. Inspired by large language models that have revolutionized natural language processing, these models are trained on massive genomic, proteomic, and structural datasets and are capable of learning complex biological relationships across multiple scales. Such models have the potential to predict protein structure, antigen presentation, immunogenicity, and immune-cell interactions simultaneously, thereby creating a unified framework for vaccine design. Rather than relying on separate algorithms for individual tasks, future vaccine development pipelines may utilize a single biological foundation model capable of integrating diverse datasets (Guo et al., 2025), and generating optimized vaccine candidates with unprecedented efficiency.

Another exciting direction involves the development of digital twins of tumors. A digital twin is a virtual computational representation of a patient’s tumor that continuously incorporates genomic, transcriptomic, and clinical information. By simulating tumor evolution and immune responses *in silico*, digital twins could enable researchers and clinicians to predict how specific tumor clones might respond to vaccination strategies before treatment is administered. Such predictive modeling could significantly improve therapeutic decision-making and facilitate the development of highly individualized immunotherapeutic interventions (Björnsson et al., 2019).

Future vaccine design pipelines are also expected to move beyond single-cell genomics toward comprehensive multi-omics integration. While single-cell sequencing provides valuable information regarding gene expression and mutational landscapes, additional layers of biological information, including epigenomics, proteomics, metabolomics, and spatial transcriptomics, can provide a more complete understanding of tumor biology. Integrating these diverse datasets through advanced AI frameworks may reveal novel biomarkers, improve neoantigen prioritization, and identify previously unrecognized mechanisms of immune evasion. Consequently, multi-omics-guided vaccine design is likely to become a cornerstone of next-generation precision oncology (Hasin et al., 2017).

Another important advancement will be the incorporation of real-time tumor monitoring through circulating tumor DNA (ctDNA) and liquid biopsy technologies. Tumors continuously evolve under selective pressures imposed by therapy and immune surveillance, resulting in dynamic changes in clonal composition. Future AI-guided vaccine platforms may utilize serial ctDNA measurements to track these evolutionary changes in near real time and update vaccine targets accordingly. Such adaptive vaccination strategies could help prevent clonal escape and maintain therapeutic efficacy throughout the course of treatment (Wan et al., 2017).

Generative AI is also expected to play an increasingly important role in the development of adaptive cancer vaccines. Current approaches typically design a vaccine based on a single snapshot of tumor data. However, future systems may continuously learn from patient-specific molecular profiles and clinical outcomes to redesign vaccine constructs as the tumor evolves. These adaptive AI-designed vaccines could provide a dynamic therapeutic response that mirrors the changing biological landscape of the disease, thereby improving long-term treatment success (Kumar et al., 2024; Katsikis et al., 2023; Kong, 2025).

There are a number of very speculative technologies at the far end of this field, and current predictive algorithms are in active experimental testing. The idea of biological foundation models, digital twins of tumors for *in silico* simulation and autonomous and adaptive AI-designed vaccines are still theoretical and in need of significant further development before it can be brought onto the near horizon of clinical use. Such systems could potentially perform the entire workflow from sequencing data analysis and neoantigen identification to vaccine optimization and clinical recommendation, with minimal human intervention. Although significant challenges related to validation, regulatory approval, data privacy, and explainability remain, these advances have the potential to transform personalized cancer vaccination from a highly specialized research endeavor into a scalable and clinically accessible therapeutic strategy. As computational and experimental technologies continue to mature, the vision of rapidly deployable, patient-specific cancer vaccines may become an achievable reality in routine oncology practice.

## 8 Conclusion

The use of multi-modal single-cell genomics and AI-based reverse vaccinology represents a promising approach to personalized cancer immunotherapy. Single-cell technologies provide a high-resolution view of intratumoral heterogeneity and dynamic clonal evolution, thereby overcoming many of the limitations associated with bulk sequencing. This detailed biological information, combined with advanced computational tools such as deep learning autoencoders for data harmonization and generative large language models for *de novo* multi-epitope formulation, has the potential to facilitate more efficient *in silico* vaccine design and the rational development of personalized vaccine candidates. Although significant challenges remain, including algorithm interpretability, data availability, rapid GMP manufacturing, tumor plasticity, and the need for robust clinical validation, continued advances in computational methods, sequencing technologies, and translational infrastructure may help address these limitations. If successfully validated in prospective clinical studies, the integration of single-cell genomics with AI-driven reverse vaccinology could become an important component of precision oncology by enabling more adaptive and individualized cancer immunotherapies. Realizing this potential will require interdisciplinary collaboration among computational scientists, immunologists, oncologists, and regulatory experts to ensure that these technologies are robust, interpretable, scalable, and clinically applicable.
